# Micro-Vibrations Analysis in LEO CubeSats Using MEMS Accelerometers

**DOI:** 10.3390/s25185917

**Published:** 2025-09-22

**Authors:** Sándor Gyányi, Róbert Szabolcsi, Péter János Varga, Gyula Horváth, Péter Horváth, Tibor Wührl

**Affiliations:** 1 Kandó Kálmán Faculty of Electrical Engineering, Obuda University, 1084 Budapest, Hungaryvarga.peter@kvk.uni-obuda.hu (P.J.V.); wuhrl.tibor@kvk.uni-obuda.hu (T.W.); 2 C3S LLC, 1097 Budapest, Hungary

**Keywords:** CubeSat, vibrations, MEMS, accelerometer

## Abstract

Small satellites or CubeSats orbiting in low Earth orbit (LEO) have become increasingly popular in Earth Observation missions, where high-resolution imaging is essential. Due to the lower mass of these spacecrafts, they are more sensitive to vibrations, and image quality can be particularly negatively affected by micro-vibrations. These vibrations originate from on-board subsystems, such as the Attitude Determination and Control System (ADCS), which uses reaction wheels to change the orientation of the satellite. The main goal of our research was to analyze these micro-vibrations so that the acquired data could be used for post-correction of camera images. Obuda University, as a participant in a research project, was tasked with designing and building a micro-vibration measuring device for the LEO CubeSat called WREN-1. In the first phase of the project, the satellite was launched into orbit, and test data were collected and analyzed. The results are presented in this article. Based on the data obtained in this way, the next step will be to analyze the images taken at the same time as the vibration measurements and to search for a correlation between the image quality and the vibrations. Based on the results of the entire project, it could be possible to improve the image quality of the onboard cameras of microsatellites.

## 1. Introduction

The advancement of precision mechanical engineering and optics, together with the increasing integration of complex data processing systems, makes nanosatellites, such as CubeSats, more and more popular in the field of Earth Observation (EO). With today’s high-end technologies, even a 3U (10 × 10 × 30 cm) CubeSat might be capable of accommodating a complete imaging system, including multiple lens, sensors, and read-out electronics, as well as increasingly sophisticated on-board data pre- or even post-processing solutions [1,2,3,4].

In the framework of Hungary’s EU-funded EO mission WREN (Water Resources in Efficient Networks), a 6U (10 × 20 × 34 cm) CubeSat called WREN-1 was built with a specific set of instruments optimized for supporting precision agriculture in Hungary from space [5]. The aim of the mission is to provide a forecast of areas at risk of drought during periods of increasingly frequent rainfall deficits and reduce drought damage in the country through drought monitoring. To observe vegetation growth, it will collect data in visible (V), near-infrared (NIR), and short-wave infrared (SWIR) wavelengths with a CubeSat-optimized multispectral push-broom imager (HICAM) and a SWIR camera module (LOCAM) originally designed for industrial applications [6].

The nature of this task makes an Attitude Determination and Control System (ADCS) inevitable, as it is essential for the optical systems to be precisely “aimed” at the areas of interest on the Earth’s surface. Because the spacecraft is traveling at approximately 7.5 km/s, the camera would sweep across the Earth’s surface too quickly; therefore, continuous camera angle adjustment is required. Because the camera is attached to the satellite in a fixed position, the ADCS provides precise, trajectory-following adjustments of the entire satellite body orientation. The most agile ADCSs use so-called reaction wheels, which are tiny flywheels driven by DC motors, so their angular momenta can be controlled electronically. As a result of the conservation of angular momentum, the orientation of the whole spacecraft can be fine-tuned and continuously managed by the on-board software. Unfortunately, this freedom in attitude control comes at a cost; the flywheels cause micro-vibrations in the spacecraft’s structure, which might affect the sensitive imaging equipment, possibly resulting in a degradation in image quality [7]. Micro-vibrations can slightly move the imaging sensor during exposure; thus, some pixels will not record the correct image information if the displacement occurs at the wrong time. This can cause varying degrees of blurring depending on the type of imaging sensor, as rolling shutter or global shutter type sensors are affected differently. Micro-vibrations in the plane of the imaging sensor can be the most disruptive, and because the orientation of the satellite can change significantly, it is necessary to measure micro-vibrations in all three axes.

To assess the severity of this problem, the WREN-1 spacecraft was equipped with a module capable of measuring the micro-vibrations of the spacecraft during operation. By analyzing these micro-vibrations and their effects on satellite images in different operational situations, we can outline recommendations for spacecraft mechanical design, develop image post-processing methods, and formalize best practices regarding spacecraft operations itself, thus ultimately improving the quality of service of CubeSat-based EO missions in general [8,9,10].

## 2. WREN-1 System Design

For the WREN-1 mission, we designed a micro-vibration sensing module, called “ÓE-IOD”, which stands for “Óbudai Egyetem—In Orbit Demonstration”. “ÓE” is the official abbreviation of Obuda University. This section contains a brief introduction to the WREN-1 spacecraft and its platform.

### 2.1. The WREN-1 Spacecraft

**Figure 1:**
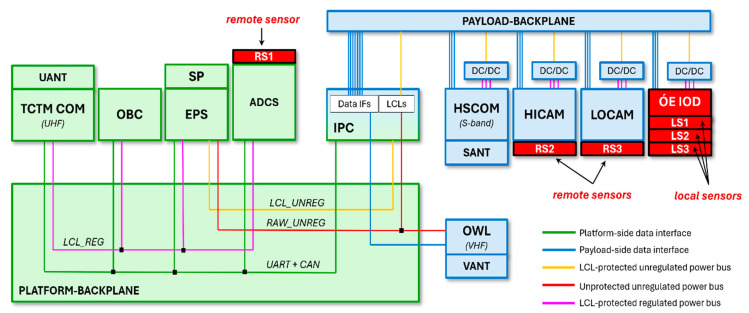
The modules and their interconnections within the WREN-1 spacecraft.

The WREN-1 spacecraft was designed and built by C3S LLC (Budapest, Hungary) in accordance with the company’s in-house CubeSat spacecraft architecture concept, depicted in Figure 1.

The spacecraft consists of two main subsystems, namely, the platform (green) and the payload (blue). The platform side includes all of the necessary modules traditionally referred to as the spacecraft “bus” or “service module”. It is responsible for providing the payload with power and making it accessible for the mission operation center through the ground station. High reliability and availability are the main properties of the platform side, which are achieved using a single-point failure-tolerant design and component-level cold redundancy [11]. The platform side comprises the following electrical modules:

- TCTM COM and UANT: The Telemetry/Telecommand Communication Module is capable of establishing radio-frequency communication between the ground station and the spacecraft. The UHF frequency band is used for both telecommand uplink and telemetry downlink. The TCTM COM module also includes two omnidirectional V-dipole antennas (UANT) [12].
- OBC: The On-Board Computer is the central element of platform-side on-board data handling. It receives telecommands from the TCTM COM and gathers telemetry from all over the spacecraft for downlink, and it is also able to manage scheduled commanding [13].
- SP and EPS: The Solar Panels are assemblies of photovoltaic solar cells capturing sunlight and converting it to unregulated DC electricity. The Electrical Power System is responsible for connecting the solar cells onto the unregulated power bus through MPPT (Maximal Power Point Tracking) circuitry, managing the charging of the secondary power source (battery), and distributing the power among the subsystems [14].
- ADCS: The Attitude Determination and Control System uses a set of sensors (e.g., sun sensors, nadir sensor, magnetometer, etc.) and actuators (magnetorquers, reaction wheels) to determine the attitude of the spacecraft and control it according to the mission’s needs [15].

The payload integration process does not place a strict and comprehensive set of requirements on payload modules from mechanical, electrical, and software points of view, as, in general, the payload modules to be integrated are pre-designed or even pre-manufactured products. C3S’ Intelligent Payload Controller (IPC) plays a “glue-logic” role in the spacecraft between the fixed platform side and the mission-specific payload side. It is basically a secondary On-Board Computer including a high-performance Linux-based System-On-Module (SoM) providing an efficient platform for on-board data processing and a mid-range FPGA, making it possible to easily adapt to the payload modules’ varying interface needs [11].

The aforementioned multispectral push-broom imager (HICAM) and the SWIR camera module (LOCAM) are the main payload modules within the WREN-1 spacecraft. As they produce large amounts of image data, a high-speed S-band downlink module was also necessary to integrate (HSCOM). The Orbital Whereabout Locator (OWL) is a VHF transmitter that periodically sends useful location information from the spacecraft, making the orbital tracking easier for the mission operators [16].

**Figure 2:**
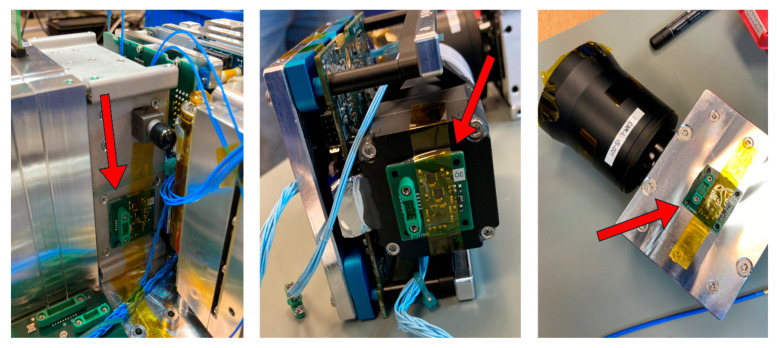
The remote sensors mounted to the ADCS, the push-broom imager, and the SWIR camera housing. Red arrows are indicating the placements of the sensors.

From a mission objective point-of-view, the micro-vibration sensing module (ÓE-IOD) plays a “secondary” (in-orbit demonstration) payload role and was built into the extra space left within the payload bay after the primary payload modules’ assemblies were finalized. There are two types of micro-vibration sensors handled by the module; the local sensors (referred to as LS1–3 in Figure 1) are placed directly on the ÓE-IOD PCB, while the remote sensors (referred to as RS1–3 in Figure 1) were mounted to the housings of the imagers and the ADCS (see Figure 2) using flexible two-component epoxy adhesive [17].

### 2.2. The Micro-Vibration Sensing Module

The ÓE-IOD module is an add-on card of the satellite system; it has its own central controller, a dsPIC33 series signal processing controller (Microchip Technology, Chandler, AZ, USA). This controller is responsible for configuring and initializing the connected electronics, as well as querying data from the acceleration sensors. Communication between theses sensors and the central controller of the ÓE-IOD is performed using two SPI interfaces. The three local sensors (named LS1 to LS3) are integrated on the ÓE-IOD module and directly connected to the SPI2 interface of the central controller as “slave” devices, while the dsPIC33 is working in “master” mode in communication. Because of the short distance between devices, transmission uses an NRZ line coding mechanism. The micro-vibration sensing module also has three external, “remote” acceleration sensors (named RS1–RS3), and communication between them uses differential signaling transmission to achieve better interference immunity and noise suppression [18,19].

**Figure 3:**
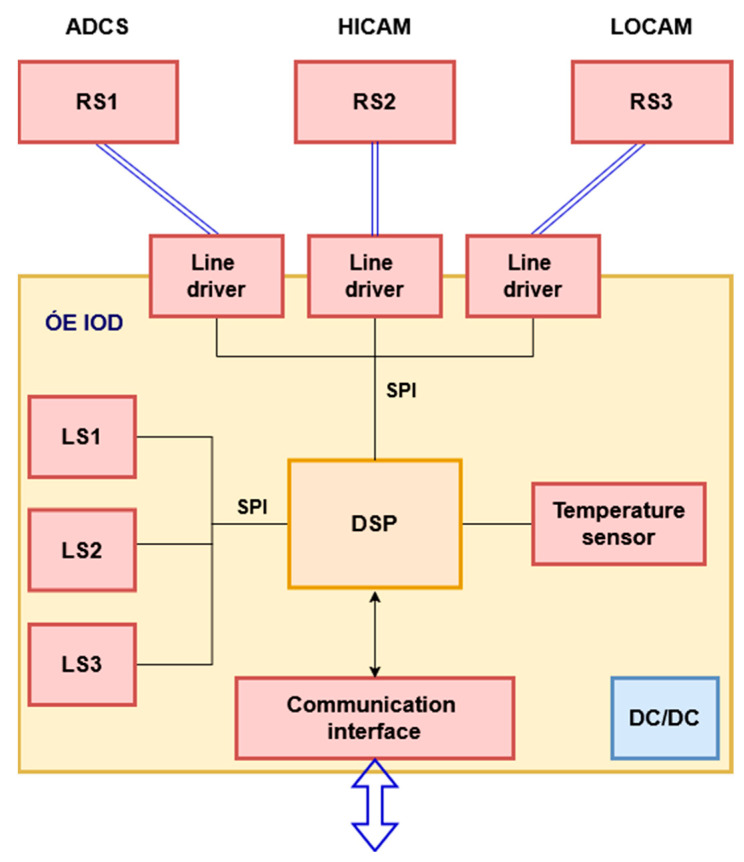
Block scheme of the ÓE-IOD module.

Figure 3 shows the block scheme of the ÓE-IOD module.

**Figure 4:**
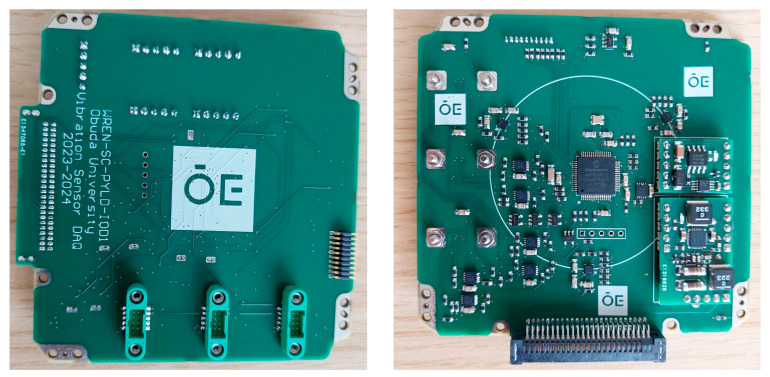
The “ÓE-IOD” module.

The main LCL-protected, unregulated power bus of the satellite provides the power supply for the ÓE-IOD module. A DC–DC converter and an overcurrent protection circuit are placed as a daughterboard onto the module, which produces stable and filtered 3.3 V supply voltage for the electronic components. Figure 4 shows the ÓE-IOD module.

Communication between the ÓE-IOD and the central controller of the WREN-1 satellite uses an UART-based communication interface. The signal level is RS422-compatible, with differential signaling. The ÓE-IOD communicates as a slave device of the satellite by using a non-standard protocol, and the power supply is switched on by the main controller of the satellite, only before the measurement starts. The measurements can be initiated to use the internal, external, or both sets of sensors. The operation starts almost instantly—within a few microseconds—and runs on all three sensors of the set simultaneously [2].

The measurements are performed autonomously by the sensors, and results are stored in an internal FIFO memory, from which the ÓE-IOD module reads them at regular intervals and temporarily stores them in its own RAM. The complete measurement data are retrieved at the initiative of the WREN-1 central controller in chunks, and the ÓE-IOD module releases the storage space after reading [20].

### 2.3. About the Micro-Vibration Sensors and Data Structure

**Figure 5:**
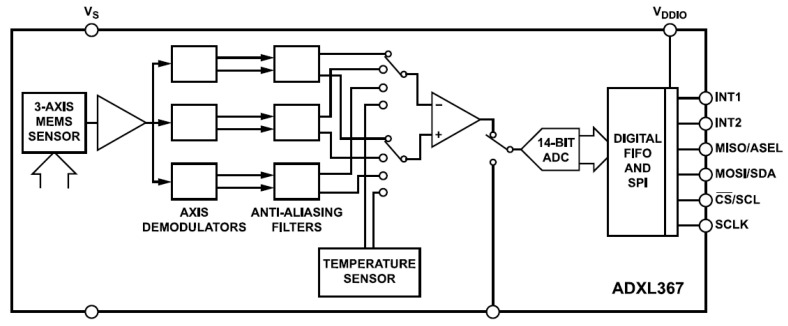
Structure of ADXL367 MEMS-based sensor [21].

For the remote and local sensors, we chose Analog Devices^®^ ADXL367 (Wilmington, MA, USA). It is a MEMS-based, 3-axis acceleration sensor with a resolution of 0.25 mg/LSB. The acceleration data are provided in an X-Y-Z coordinate system. Reasons for choosing this sensor are as follows: it is small, it has very low power consumption, it has a fast SPI communication interface, and it can measure acceleration on 3 axes simultaneously. We assumed that it could survive the harsh conditions on board the satellite, and it did. It has an error detection mechanism to detect accidental register content changes due to external influences; our tests later confirmed that various radiations did not cause such errors. The structural design of the sensor is shown in Figure 5 [21]:

For the measurement, the following configuration was used:

- FIFO mode, where an interruption is generated after 20 × 3 samples, with 20 samples in all 3 axes.
- 100 sample/s rate.
- ± 2 g range.
- Number format is 14 bits + 2 axis identification bits.

The measurement algorithm is the following:

- After the power supply is switched on, the program makes an initialization process and resets the necessary interface devices.
- It resets the acceleration sensors and configures the necessary registers after performing a soft-reset process.
- The microcontroller goes to standby mode and waits for measurement instructions from the on-board controller.
- After the measurement command is received, the acceleration sensor measurement process starts, and the FIFO buffers will be filled with measurement data. The first measurement data are generated after 10 ms + 20 µs.
- After the twentieth sample arrives in the acceleration sensor FIFO buffer, the device sends an interrupt request to the microcontroller, which in turn queries and reads the data stored in the sensors’ FIFO buffers. During the readout, the sensors continue to perform measurements autonomously. During a readout process, 120 bytes are transferred (20 sample packets, with 3 axis data per packet, where one sample is 16 bits in size).
- The readout uses the corresponding SPI bus. Both SPI buses’ clock is 1 MHz, meaning the readout speed is 1 Mbit/s. The readout time of the entire acceleration sensor FIFO is therefore 120 × 8 × 1 µs = 960µs per sensor [21].

All acceleration data elements are stored in 16 bits, and the number format is the following:

- [b15, b14]: axis identifier bits (00: X axis; 01: Y axis; 10: Z-axis).
- [b13–b0]: 14-bit signed integer acceleration value, where b13 is the MSB.

The order of axes in the FIFO buffer: X, Y, Z.

Sampling is performed at 10 ms intervals, i.e., the sampling frequency is 100 Hz. The built-in low-pass filter of the sensor prevents spectrum overlap, so, in theory, the acceleration sensor can detect harmonic vibration patterns of up to 50 Hz in frequency.

Communication between the satellite’s main controller and the ÓE-IOD vibration measurement panel uses its own packet format. An 8-byte data packet is used to start the measurement, and the first byte is the command identifier. The response messages use a uniform packet structure, and each packet is 128 bytes in size [22]. The response structure looks like this:
+0: 0x81”Response to a specific command”+1Sensor ID (from 0x01 to 0x06)+2–+121Sensor data (20 × 6 bytes)+122, +123Sequence number+124Not used+125Not used+126Not used+127:Checksum

Because a complete measurement usually generates a larger amount of data, which cannot fit in a single packet, each packet is transferring only a chunk of the measurement results. Each packet has a sequence number, which can be used for reassembly of the data set. An 8-bit checksum helps to detect data corruption and transmission errors.

After the query, the main controller inserts a time stamp into the packets, which allows for the determination of the time of the measurements. The ÓE-IOD controller does not use internal time stamps, but the measurement series contains the measured values without interruption after the start, so the time of generation of each sample can also be identified.

**Figure 6:**
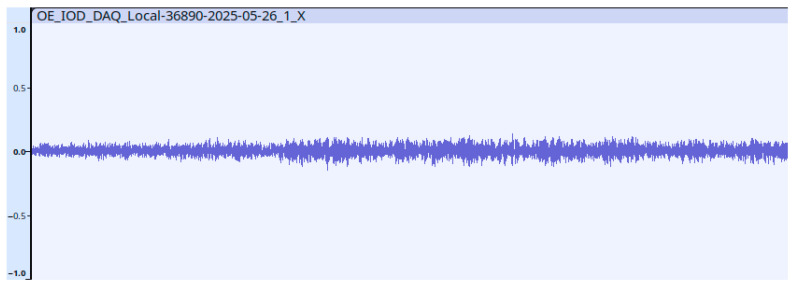
Vibration data in time domain displayed by Audacity.

During the pre-processing of the vibration data, a conversion program developed by us generates a WAV (Waveform Audio File Format) file for X, Y, and Z axes of each sensor for easier further processing. In the WAV file header, we store the data format (1 channel, 16-bit resolution, PCM type) and the sampling frequency (100 Hz) so that processing can be performed with practically any application that is compatible with the WAV format [23,24]. Figure 6 shows the vibration data in the time domain displayed by Audacity.

**Figure 7:**
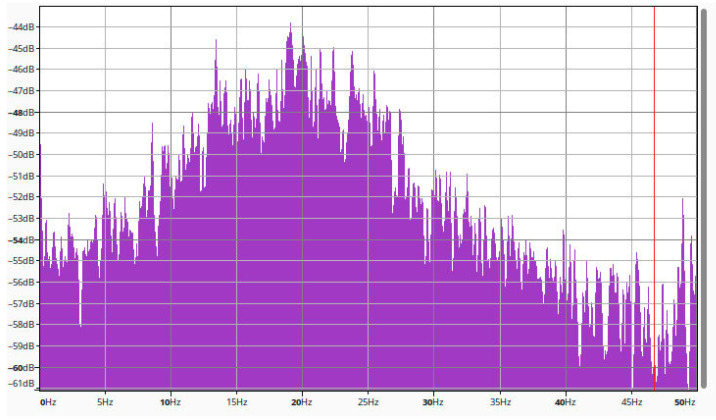
Fast Fourier Transformation of vibration data, displayed by Audacity.

Some of these tools can be used for making basic transformations, like Fast Fourier Transformation. Figure 7 shows the Fast Fourier Transformation of vibration data, displayed by Audacity.

## 3. Telemetry Analysis Results

In this section, we discuss the results of processing the captured data. We obtained acceleration data in orbit, with ADCS on and ADCS off states.

### 3.1. Data Structures and Conversion

The data were processed in the MATLAB (R2025a) environment, because this environment has many built-in tools for signal processing. The first step of processing was to read the files containing the vibration data stored in WAV format. Although the primary application of the WAV format is to store audio data, the amplitude of the captured acceleration data samples can be found in the file in the same INT16 format, and the sampling frequency is also stored. MATLAB has a built-in function to read WAV format files into an “N” element vector and returns the sampling frequency as a variable [25].

**[accelerationData, fs] = audioread(wavFile, ‘native’);**

The MATLAB code above uses the “audioread” function, which returns a vector named “accelerationData” of length “N” and a variable named “fs” containing the sampling frequency value. Because the acceleration sensor produces 14-bit values, the INT16 format numbers in the vector actually contain 14 significant bits only, and the lower two bits of the 16-bit number always carry the value ‘0’. The number representation is signed in two’s complement format.

The full range of acceleration data samples is 4 g, where the positive maximum number represents +2 g, while the lowest negative value is −2 g. The data in the vector in this processing step are not yet suitable for direct mathematical processing, as they do not represent a physical quantity. For conversion, the measurement values must be converted to floating-point fractions and then converted to m/s^2^ values. Because the entire number range represents acceleration values between −2 g and +2 g in 16-bit resolution, the entire 4 g range is divided into 65,536 steps. It is important to mention that the “Z” axis of the acceleration sensors works on a different principle than the “X” and “Y” axes. This axis is compensated for when the Earth’s gravity at rest; therefore, this axis indicates a value of 1 g when no acceleration is sensed in this axis. This effect should be compensated for during processing [26]. Based on these calculations, conversion can be performed by using the following MATLAB code:

**g = 9.80665;**

wav = (double(accelerationData) * 4 * g/65,536) − zBias;

The “zBias” variable contains 1 if the code is processing the “Z” axis and 0 for the other axes.

### 3.2. Theory of Data Processing

The acceleration data stored in our “wav” vector are in units of [m/s^2^]. Our goal is to calculate displacement data—or spatium—from these data, which in theory can be achieved through double integral calculation of the acceleration data with respect to time. By integrating the acceleration values with respect to time, we obtain the velocity changes, and by integrating the velocity vector with respect to time, we obtain the displacements. Because the measurements could start at any moment in time, the measurement system does not have any prior information about the current speed or position of the entire unit; therefore, we have to calculate with zero initial velocity and zero displacement. The acceleration sensors measure the acceleration values relative to the starting position, so the measured values will be interpreted relative to these zero points [27,28].

At first, we calculated the trapezoid integral by writing our code, but MATLAB does have a built-in function that can calculate the trapezoid integral of the elements of a vector and accumulate the resulting values. This function can utilize the GPU, making the calculation much faster.

**Figure 8:**
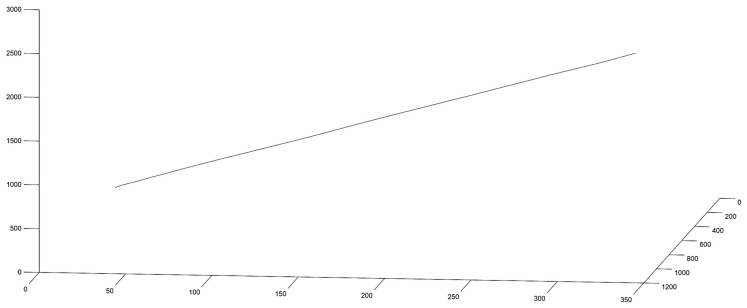
Movement of the object in 3D.

During the evaluation of the results, the issue of low-frequency acceleration components had to be resolved, because the project aims for a micro-vibration analysis. Because the satellite is in a low Earth orbit (LEO), the circumferential motion, the ADCS, and the Earth’s gravity have continuous effects on the device, which generate acceleration of a larger amplitude than the vibrations generated by the onboard instruments of the spacecraft. The global motion calculated from the measured acceleration values therefore suppresses the values meant to be measured. We cannot subtract low-amplitude acceleration values from the measured values for applying simple compensation because their direction and their effect on the different axes are not constant [29,30]. Figure 8 shows the movement of the object in 3D.

According to the evaluated data, the displacement—calculated by using the acceleration data—was between 340 and 2515 m, depending on the direction of the accelerometer axes and the current orientation and position of the satellite. Table 1 shows the example displacement values from measurement data.

**Figure 9:**
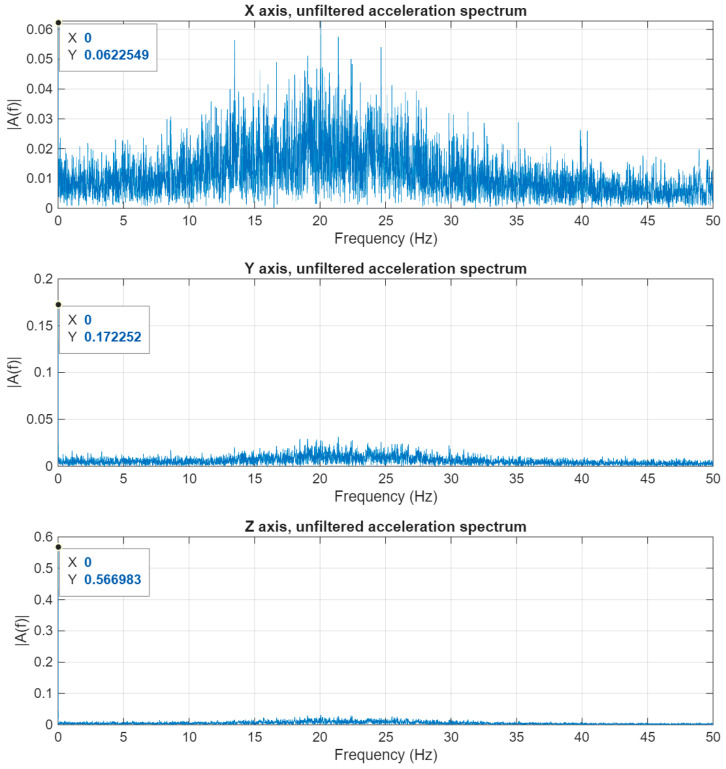
Three-axis acceleration data in frequency domain.

Examining the measured acceleration values on the different axes, it is evident that on two of the three axes—Y and Z—there is a low-frequency acceleration value, and its amplitude is significantly larger than the values measured on the third X-axis. The two low-frequency components contain the acceleration values of the satellite’s angular motion and its change in motion towards the Earth. These components are considered disturbances from the point of view of micro-vibrations. Figure 9 shows the spectrum of the accelerations measured on the three axes.

**Figure 10:**
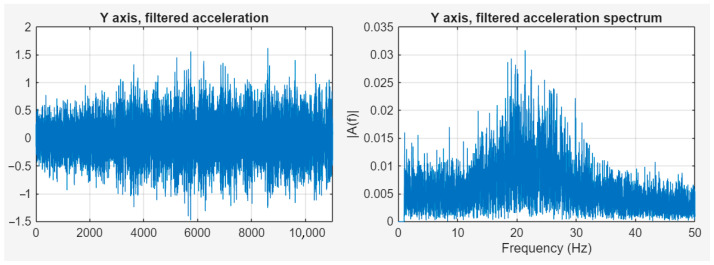
Filtered signal in time and frequency domains.

To examine vibrations, it is necessary to get rid of the very low frequency components. After examination of several methods, the most promising solution was component removal in the frequency domain data generated by the Fast Fourier Transformation. This process starts with the FFT; the components to be suppressed in the frequency domain must be identified, and their amplitude must be reduced, even to zero. After this operation, the signal could be transformed to the time domain without the unnecessary frequency components. Although such a drastic suppression may cause the Gibbs phenomenon, in the experiment, when the signal transformed back to the time domain after the 0 Hz component was zeroed, this effect was not present. However, the case would be different if the zeroing operation involved higher frequency components [31,32,33]. Figure 10 shows the filtered signal in the time and frequency domains.

### 3.3. Vibration Data Analysis with ADCS off

By assumption, a significant part of micro-vibration generation comes from the ADCS (Attitude Determination and Control Systems), as a mechanical, rotating system.

**Figure 11:**
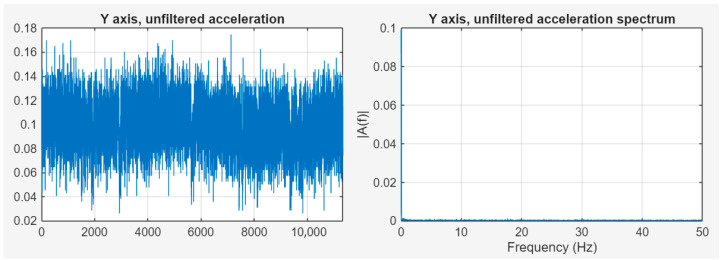
ADCS wheel off; unfiltered acceleration data in time and frequency domains.

The measurements were performed with the ADCS on and off. With the ADCS off, the measured data show that there is practically no significant, identifiable frequency vibration component that would affect the imaging of the on-board camera. The spectrum contains a spike around 0 Hz, which is due to the global movement of the object [34,35]. Figure 11 shows the ADCS wheel off, with unfiltered acceleration data in the time and frequency domains and filtered signals in the time and frequency domains.

The most suitable state for taking photographs would be with the ADCS turned off; however, in this case, the camera position cannot be positioned to the appropriate target [36,37]. The movement of the satellite would cause very significant viewing angle distortion, resulting in image blurring effects.

### 3.4. Vibration Data Analysis with ADCS on

**Figure 12:**
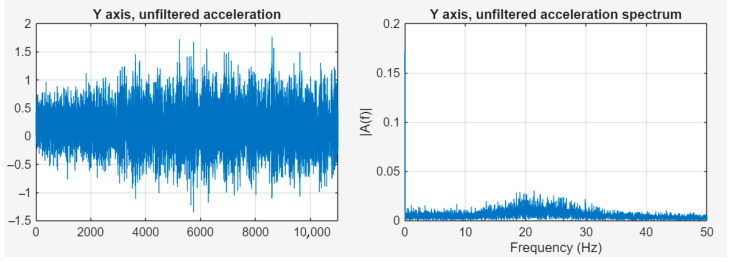
Acceleration data with ADCS on, unfiltered, in time and frequency domains.

In order to position the camera precisely, the ADCS operated in “Tracking” mode, i.e., it corrected the satellite’s orientation around its own axis so that the lower side containing the camera was always pointed at the targeted point. This required spinning the ADCS flywheel, which naturally caused vibrations [38,39]. Figure 12 shows the acceleration data with ADCS on, unfiltered, in the time and frequency domains.

**Figure 13:**
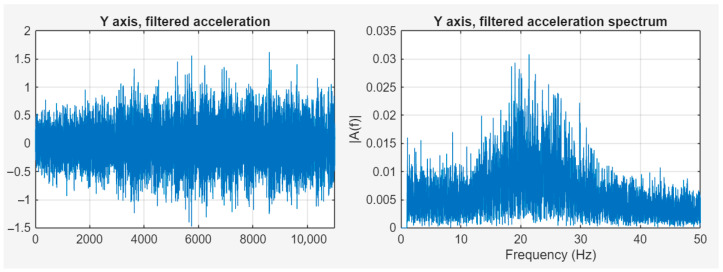
Acceleration data with ADCS on, filtered, in time and frequency domains.

The figure above shows that there is a significant frequency component around 21.5 Hz—in the same range as the acceleration values affecting global movement of the satellite—which can clearly be linked to the switched-on state of the ADCS. Figure 13 shows the acceleration data with ADCS on, filtered, in the time and frequency domains.

By removing the components between the 0 and 1 Hz range, the spectrum image does not change significantly, as the amplitude of the vibration falls in the same range as the low-frequency acceleration. By examining the path calculated based on the measured and filtered accelerations, i.e., the actual vibration, a spatial image of the physical impacts on the camera can be determined.

The vibration data obtained in this way could be suitable for modeling the movement of the imaging sensor during the camera’s exposure, which can be used to improve image quality during post-processing [40,41,42].

### 3.5. An Application Example for CubeSat Designers

In the previous sections, we presented the presence and spectrum of micro-vibrations caused by ADCS. There are multiple possible solutions to improve the quality of the image captured by the camera placed on the CubeSat. One solution could be a post-processing algorithm performed on the captured image by using the vibrations’ timing and amplitude values. Using another approach, harmful resonances can be filtered out during the designing and building phase with mechanical vibration damping elements tuned to the appropriate frequency.

As an example, we would like to suggest a possible implementation of the previously mentioned filter. In the following example, we used the attributes of the HIRES camera located on the WREN-1 satellite. The satellite orbits the Earth at an altitude of approximately 500 km, and the GSD of the camera is 16 m at this altitude. For the calculations, we assume that the optics have a focal length of 40 mm. A 1 micrometer displacement in the sensor plane causes a 12.5 m difference in the image of the Earth’s surface, according to the following relationship:

Earth displacement = Sensor displacement × Altitude/Focal length

Based on our data, the maximum amplitude of the ADCS-induced displacements and unwanted deviations caused by micro-vibrations were around 5 μm, which can cause a displacement of 3–4 pixels if the exposure occurs at the wrong moment. The effect may depend on several factors, including the exposure time, the sensor type, and the exposure method. By filtering out the components causing these significant deviations from the ADCS-induced vibration spectrum, these unwanted displacements can be significantly suppressed by a properly tuned mechanical filter. To determine the transfer function of this structure, we analyzed the vibration spectrum as a case study using MATLAB. For implementing a low-pass filter, we first determined a first-order transfer function in the following form:

$$H\left(s\right)=\frac{K}{1+\tau \cdot s}$$

In this function, *K* is the gain factor; its value should be as small as possible, as this means frequency-independent attenuation. In the case of purely frequency-dependent attenuation, this value is 1. *τ* is the time constant of the system, which is related to the cutoff frequency ($f_{c}$):

$$\tau =\frac{1}{2\cdot \pi \cdot f_{c}}$$

As Figure 13 shows, when the ADCS is on, the amplitudes of the micro-vibrations increase significantly above 15 Hz. The low-frequency movements are mainly caused by orbital movements.

We chose 10 Hz as the cutting frequency in the case study. The following MATLAB code snippet was used for processing:

**[xb,xa] = butter(1, 0.2, ‘low’);**

**filtered_s = filter(xb, xa, s);**

For the camera suspension, the description of the suspension system can be approximated by a quadratic transfer function. The transfer of the quadratic low-pass filter can be described by the following equation:

$$H\left(s\right)=\frac{K}{s^{2}+2\cdot \xi \cdot \omega_{0}\cdot s+\omega_{0}^{2}}=\frac{\frac{K}{\omega_{0}^{2}}}{1+\frac{2\cdot \xi }{\omega_{0}}\cdot s+\frac{1}{\omega_{0}^{2}}\cdot s^{2}}$$

In the equation above, $\omega_{0}\;$ is the natural resonance and *ξ* is the attenuation factor, which should be less than 1 in this application (an overdamped case) to avoid resonances.

The vibration suppression simulation can be performed as follows:

**[xb,xa] = butter(2, 0.2, ‘low’);**

**filtered_s = filter(xb, xa, s);**

**Figure 14:**
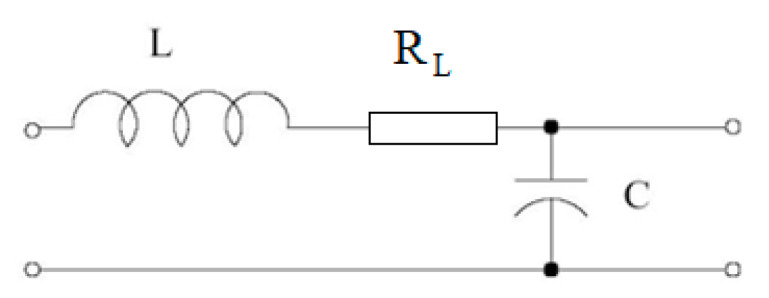
Electronic model of a second-order filter.

The transfer function of the filter circuit as shown in Figure 14 is the following:

$$\left(s\right)=\frac{1}{L\cdot C\cdot s^{2}+\frac{L}{R_{L}}\cdot s+1}$$

Self-resonance of the filter: ω0=1LC

The attenuation factor: ξ=12·RL·LC

For calculating the camera suspension mechanism model, we used the following analogy: *L* ≡ *m*; *C* ≡ 1/*k*; *b* ≡ $R_{L}$.

Based on this model, the transfer function of the mechanical system is the following:

$$H\left(s\right)=\frac{1}{\frac{m}{k}\cdot s^{2}+\frac{b}{k}\cdot s+1}$$

In the mechanical suspension system, “*m*” is the mass of the camera, *k* is the spring constant, and $R_{L}$ is the hysteretic damping. These two values describe the mechanical parameters of the mechanical suspension system.

**Figure 15:**
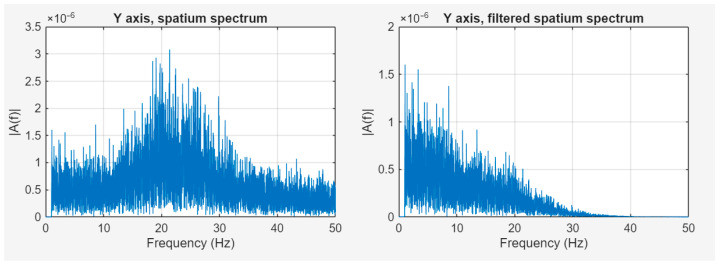
Comparison of the unfiltered and filtered camera displacements.

The goal of this mechanical filter is to significantly reduce the effects of the ADCS-generated micro-vibrations. Figure 15 shows the spectrum of camera displacement before and after filtering based on our model. The displacement values in the critical 20–30 Hz range were significantly reduced by the filter.

## 4. Discussion

The use of LEO satellites is becoming increasingly popular today, which is why they are widely used in multiple fields of science and engineering. The possibilities of use and application are also expanding in communication and photo or video recording. In many cases, these satellites do not carry propellant on board, so the positioning and orientation controls are performed with the help of ADCS using the gyroscopic principle. Mechanical movement—like the flying wheel rotation—on board causes micro-vibrations, which can affect the usability of the spacecraft and the quality of the photographs and video recordings taken by the satellite. The analysis of these vibrations can provide useful information for designers and platform builders of LEO satellites, and the availability of micro-vibration time series data can provide useful image processing information for subsequent image corrections. The captured data from the MEMS acceleration sensors placed on the WREN-1 satellite contain acceleration data caused by both the arc trajectory and micro-vibrations. In this article, we have published results obtained from the satellite on-board sensor module, and we propose an algorithm to remove the acceleration values caused by the trajectory. The remaining vibration data obtained in this way can provide useful information for image processing algorithms, such as, for example, through correlation analysis of image noise originating from vibrations, which can help to reduce image noise.

The captured acceleration data contains input from multiple sensors, which can be used for further analysis. The local sensors (LS1, LS2, and LS3) are placed at an angle of 120 degrees along a circle on the ÓE-IOD motherboard, and the data contain samples from these sensors captured at the same time. These correlated data could be the basis for examining the 3D micro-movements of the whole ÓE-IOD board. Our article does not deal with the latter options, but we publish pre-processed WAV files 110 s in length for further free analysis and further research in the section “Supplementary Materials”.

## 5. Conclusions

In a small satellite, MEMS-based acceleration sensors can provide a variety of useful information by recording instantaneous acceleration data on board the spacecraft. The time series generated from the acceleration data can be efficiently transformed using a variety of signal processing methods, such as, for example, in the MATLAB^®^ environment or a real-time DSP device. This data analysis can be useful for CubeSat designers and builders because it can provide correction parameters for image post-processing algorithms. These data also can help to design flexible, properly tuned suspensions to suppress unwanted vibrations and thus their effects on board.

In our article, we examined the micro-vibrations generated by ADCS on board an operational satellite, and we measured them with MEMS-based acceleration sensors. We processed the recorded data and, based on the results, we designed a filter model that provides tuning parameters for designing and implementing a mechanical suspension that will reduce the effects of the vibration.

## Figures and Tables

**Table 1:** Example displacement values from measurement data.

Axis	Displacement in Meters
X	360
Y	1086
Z	3456

## Data Availability

Further inquiries can be directed to the corresponding author.

## Supplementary Materials

The following supporting information can be downloaded at https://drive.hti.kvk.uni-obuda.hu/s/emvbDFn2AZHprJb (accessed on 15 September 2025).

## Abbreviations

The following abbreviations are used in this manuscript:

ADCS	Attitude Determination and Control System
DSP	Digital Signal Processing
EO	Earth Observation
FFT	Fast Fourier Transform
FIFO	First In, First Out
IPC	Intelligent Payload Controller
LEO	Low Earth Orbit
MEMS	Micro-Electro-Mechanical Systems
NIR	Near-Infrared
NRZ	Non-Return-to-Zero
OBC	On-Board Computer
OWL	Orbital Whereabout Locator
PCM	Pulse Code Modulation
SWIR	Short-Wave Infrared
TCTM	Telemetry/Telecommand Communication Module
WAV	Waveform Audio File Format
WREN	Water Resources in Efficient Networks
